# Exploring the Dose-Response Relationship between Resistance-Training Volume and Psychophysiological Responses in Trained Males

**DOI:** 10.5114/jhk/209051

**Published:** 2025-11-19

**Authors:** Alysson Enes, Guilherme Nass, Ragami C. Alves, Mohan Adam E, Alec Piñero, Gustavo Oneda, Danilo Fonseca Leonel, Brad J. Schoenfeld, Tácito P. Souza-Junior

**Affiliations:** 1 Metabolism, Nutrition and Strength Training Research Group, Federal University of Paraná (UFPR), Curitiba, Brazil.; 2 Applied Muscle Development Laboratory, Department of Exercise Science and Recreation, CUNY Lehman College, Bronx, NY, USA.; 3 Sports Center, Department of Physical Education, Federal University of Santa Catarina (UFSC), Florianópolis, Brazil.; 4 Athletics and Endurance Runners Research Group (PACE), Department of Physical Education, Federal University of Jequitinhonha and Mucuri Valleys (UFVJM), Diamantina, Brazil.

**Keywords:** session rating of perceived exertion, feelings of pleasure/displeasure, training volume, volume load, strength training

## Abstract

We investigated the effects of different weekly-set progressions on perceptual responses and explored whether muscle thickness (MT) responses were related to perceptual and volume metrics in resistance-trained males. Thirty-one participants were randomly assigned to one of the three groups: a fixed set (FS) group (n = 10), maintaining 22 weekly quadriceps sets; a 4-Set (4S) group (n = 10) and a 6-Set (6S) group (n = 11), increasing by four and six every fortnight, respectively. Training occurred twice weekly for 12 weeks. The session rating of perceived exertion (sRPE) and session feelings of pleasure/displeasure (sFPD) were assessed 15 min post-training, while sRPE-derived metrics (monotony and strain) were analyzed weekly. The sRPE was higher in the FS compared to the 4S group (p = 0.046), while sFPD were higher in the FS compared to the 6S group (p < 0.001). Monotony was lower in the 4S than in other groups (p < 0.001). Strain followed a graded dose-response (6S > 4S > FS, p < 0.001). Overall, there were no meaningful correlations between volume load slopes and MT (r < 0.392) or perceptual responses (r < 0.448). These findings suggest that progressive increases in weekly-set volume influence perceptual responses; however, these responses do not appear to be related to changes in muscle thickness.

## Introduction

Resistance training (RT) volume can be quantified as either the number of repetitions, the volume load (i.e., the product of sets × repetitions × load) (VL), or the weekly set number per muscle group (Figueiredo et al., 2018; Schoenfeld et al., 2021). Current research suggests differential adaptive responses between RT volume and muscular adaptations (Baz-Valle et al., 2022; Schoenfeld et al., 2017), which could be due to potential confounding factors and methodological differences among studies such as training status, previous RT volume, and the amount of sets per week. Likewise, the number of sets compared within a single intervention can vary widely (Baz-Valle et al., 2022; Krieger, 2010; Scarpelli et al., 2020). In addition, it has been suggested that high-volume protocols may be less beneficial due to the greater extent of performance decrements (Bartolomei et al., 2017; Da Silva et al., 2020; Walker et al., 2011, 2012), which can impair recovery and hinder readiness for subsequent sessions, potentially diminishing the RT-induced muscular adaptations (Sousa et al., 2024).

Some objective fatigue markers are invasive and expensive, making low-cost, non-invasive options like subjective (i.e., perceptual) metrics a potentially more viable approach for monitoring training (Scott et al., 2016; Sousa et al., 2024). Although these methods have inherent limitations, perceptual markers offer an effective and practical means for continuous monitoring of the dose-response relationship between RT stimuli and the desired outcomes (Helms et al., 2020; Scott et al., 2016). The session Rating of Perceived Exertion (sRPE) method is commonly used to monitor the perceptual responses to RT (Helms et al., 2020; Scott et al., 2016). In addition, sRPE-derived metrics such as training monotony, calculated by the weekly sRPE values divided by the standard deviation, and strain, the product of the weekly sRPE and monotony, are related to functional and morphological adaptations (Conlon et al., 2018; Foster, 1998; Foster et al., 2001; Haddad et al., 2017; Helms et al., 2020; Scott et al., 2016). Both metrics are related to potentially negative training adaptations such as a high incidence of illness and poor performance (Foster, 1998; Foster et al., 2001; Haddad et al., 2017; Putlur et al., 2004; Scott et al., 2016). Theoretically, a constant exposure to elevated training loads, tracked by heightened perceptual responses, may lead to the onset of nonfunctional overreaching, which could potentially turn in lack of RT-induced adaptations (Foster, 1998; Foster et al., 2001; Haddad et al., 2017; Scott et al., 2016).

Previous studies have investigated the influence of RT volume on perceptual responses (Aube et al., 2022; Enes et al., 2024; Lodo et al., 2012; McGuigan, et al., 2012). These studies investigated the effects of RT volume calculated by the weekly-set number in resistance-trained individuals (squatting at least 1.5 times participants’ body mass) (Aube et al., 2022; Enes et al., 2024) and the volume load in recreationally-trained males (at least 6 months of RT experience) (Lodo et al., 2012). The former studies (Aube et al., 2022; Enes et al., 2024) were interventional protocols that lasted eight weeks with trained cohorts comparing different weekly-set numbers, and they did not observe any influence of RT volume on perceptual responses. The latter study (Lodo et al., 2012) was performed with acute RT bouts, and those authors observed a moderate correlation between the volume load lifted in the bouts and their respective sRPE. Importantly, studies that kept the weekly-set number fixed over the intervention or those that only studied acute effects limit the ability to draw conclusions regarding a possible linear dose-response relationship between the external training load (i.e., RT volume) and perceptual responses. Indeed, while there is an established linear relationship between RT intensity, training load, and both physical and emotional stress (e.g., feelings of pleasure/displeasure) (Genner and Weston, 2014; Martorelli et al., 2021; Scott et al., 2016), the linearity of this relationship with RT volume remains uncertain. Although the sRPE method is a useful and efficient tool for RT monitoring (Gomes et al., 2020), its relationship with hypertrophic adaptations remains unclear. While optimal hypertrophy may be attained by employing high-effort training routines (Refalo et al., 2021, 2023), such a level of effort may influence sRPE responses (Refalo et al., 2023), and indeed the linearity of the sRPE-muscular hypertrophy relationship has not been thoroughly investigated. Consequently, it remains uncertain whether the sRPE can reliably predict hypertrophic outcomes throughout a training block.

Therefore, we aimed to investigate the effects of different weekly set progression schemes on perceptual responses in resistance-trained males. We hypothesized a linear relationship between RT volume and perceptual responses due to the increases in the external training load (weekly sets). In addition, we investigated whether potential variables (muscle thickness (MT) and volume-load progression) might be related to the perceptual responses in an exploratory analysis.

## Methods

### Participants

Forty-three resistance-trained males volunteered to participate in the intervention. This study was part of a larger research project that investigated the effects of weekly-set progressions on muscular adaptations. Therefore, we calculated the sample power *a priori* in G*Power (version 3.1) for F family using a repeated measures analysis of variance (ANOVA) within-between interaction to determine the number of participants required to achieve a proper statistical power for muscle hypertrophy. The analysis performed *a priori* with an alpha of 0.05, power 0.80, a moderate effect size *f* of 0.25 and correlation among repeated measures of 0.7 indicated that 27 participants would be sufficient to achieve adequate statistical power (Enes et al., 2023a). Although we did not perform our calculations based on perceptual responses, a previous study with a smaller sample size, participants with similar characteristics and comparing training volumes detected a main effect of time for perceptual responses (Enes et al., 2024).

Our inclusion criteria were as follows: i) males aged 18–30 years with at least two years of RT exercising four days per week; ii) negative answers to all Physical Activity Readiness Questionnaire items; iii) minimum barbell back squat relative strength of 1.5 x body mass; iv) no creatine supplementation for at least 6 months prior to the study commencement; and v) self-reported to be free from the use of anti-inflammatory, adrenergic and/or anabolic androgenic steroid drugs. Participants were excluded if they reported any musculoskeletal injury and/or alcohol/drug abuse. Additionally, if a participant did not take part in at least 90% of the training sessions or were deemed to have performed additional resistance training outside of the supervised protocol, their data were excluded from statistical analysis.

**Figure 1:**
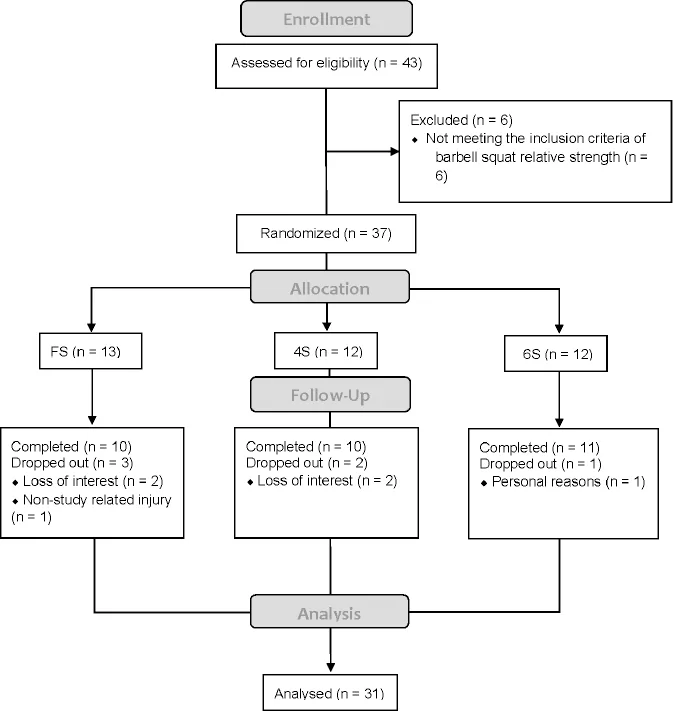
CONSORT flow diagram. Note: FS: fixed sets; 4S: four set progression training volume; 6S: six set progression training volume

All participants were informed of the procedures and specifics of the training intervention and signed a written informed consent form prior to their participation. After the initial phases, 37 participants were allocated to one of the three groups: FS (n = 13), 4S (n = 12), or 6S (n = 12), based on their dominant vastus lateralis cross-sectional area from the largest to the smallest, with the first three participants randomly assigned to one of the experimental groups, followed by the next three and so on. The outcomes regarding changes in the vastus lateralis cross-sectional area had been previously published (Enes et al., 2023a). After the intervention, thirty-one participants (age 24.4 ± years; body height 175.5 ± 6.5 cm; body mass 80.1 ± 9.4 kg; body fat percentage 14.4 ± 3.1; RT experience 5.1 ± 2.2 years; 1RM barbell back squat: body mass ratio 1.7 ± 0.1) finished the intervention (FS, n = 10; 4S, n = 10; 6S, n = 11). The dropout reasons were related to loss of interest (n = 4), personal reasons (n = 1) and non-study related injury (n = 1). The CONSORT diagram (Figure 1) depicts the details of these stages. The remaining participants attended all 24 training sessions. All procedures were approved by the ethics committee of the Federal University of Paraná (UFPR), Curitiba, Brazil (protocol code: 57870122.2.0000.0102; approval date: 10 June 2022) and were in accordance with the Declaration of Helsinki.

### Study Design

This study was a randomized, parallel-group, repeated measures trial designed to investigate the effects of different RT-volume progression models in resistance-trained males. As mentioned, the study was part of a larger research project from our laboratory and none of the analyses herein had been previously reported; however, anthropometric data as well as strength and hypertrophy outcomes had already been published (Enes et al., 2023a). Before the study commencement, MT was assessed. Afterwards, this study had a three-phased experimental design. After baseline assessments and based on the previous two-week self-reported resistance training log for quadriceps, in the first phase, we reduced the participants’ previous quadriceps volume for two weeks, reducing it by 40% every week over this period. The second phase involved a familiarization stage over another two-week period, during which participants increased their quadriceps set volume by 4 weekly sets for the fixed sets group (FS) and 4-Set group (4S), and 6 weekly sets for the 6-Set group (6S) to be acclimatized to the volume progression. Thereafter, participants entered the intervention phase in which all training groups started the training program at 22 sets per week targeting the quadriceps femoris. During the intervention, the FS group maintained a volume of 22 weekly sets targeting the quadriceps, while 4S and 6S groups progressively increased their weekly set volume by 4 and 6 sets per week, respectively, every two weeks, reaching 42 (4S) and 52 (6S) quadriceps sets per week, respectively, in the last two weeks. Participants performed training sessions twice a week with the same training routine (i.e., one loading scheme, rest intervals, etc.), differing only in RT volume quantified by weekly sets for the quadriceps. All the perceptual responses were assessed 15 min after the end of each training session. During baseline testing, participants received instructions about the perceptual scales. The volume load (VL) was calculated by the product of sets × repetitions × load (kg) for quadriceps exercises (barbell back squat, 45^o^ leg press and seated knee extension) throughout the 12-week period (24 training sessions). MT was measured at least 72 hours after the last training session.

### Procedures

#### Anthropometry

Participants underwent anthropometric and body composition assessments before the intervention to obtain baseline characteristics. A multifrequency bioelectrical impedance analyzer (InBody 120; BioSpace Inc., West Des Moines, IA) was used to determine body mass and the body fat percentage. Body height was measured using a stadiometer. Participants were instructed to adhere to the procedures given by manufacturers, such as: i) no alcoholic and caffeinated beverages consumed 24 h prior to and no food or water consumption 2 h prior to the measurement; ii) no moderate to vigorous physical activity for 72 h prior to the measurement; and iii) if possible, empty the bladder by urinating 30 min before the measurement.

#### Muscle Thickness

Lateral thigh MT was assessed using a B-Mode ultrasound (ECO3, Chison Medical Imaging Ltd., Jiang Su Province, China) with a 5-MHz linear transducer. Ultrasound-derived muscle thickness (MT) has been shown to strongly correlate with gold-standard measures such as the magnetic resonance imaging (MRI) cross-sectional area, while also offering high reliability and lower cost compared to MRI, making it a practical and reliable tool for assessing hypertrophic changes in longitudinal studies (Franchi et al., 2018). Participants were instructed to refrain from engaging in any moderate to vigorous physical activity for at least 72 h prior the first lab visit (for anthropometry and ultrasound imaging) to avoid potential confounding factors from muscle swelling/edema.

The image acquisition was acquired with participants lying supine on a stretcher, with relaxed joints and knees slightly flexed in the dominant limb, determined by asking participants which limb they would use to kick a ball. MT was analyzed at 50% of the femur length, considered as the distance from the greater trochanter to the lateral condyle of the femur. A trained ultrasound technician, blinded to group allocation, applied a generous amount of water-soluble transmission gel to the measurement site. Images were acquired transversally by placing the linear transducer against the skin taking care not to depress the skin. When the image was deemed suitable, it was saved in the ultrasound unit’s hard drive for further analysis. MT was measured as the distance between the internal border of the superficial aponeurosis of the vastus lateralis and the external border of the femur, comprising a composite of the vastus lateralis and the vastus intermedius. Three images of this site were recorded, and measurements were averaged to obtain a final value. If one of the three images differed by more than 10%, a fourth image was made and the furthest value was replaced. As previously reported (Enes et al., 2023a), the intraclass correlation coefficient, standard error of measurement, the coefficient of variation and minimal detectable difference were 0.99, 0.04 cm, 0.56% and 0.09 cm for this measurement at this site, respectively. The vastus lateralis cross-sectional area and the sum of MT of the proximal, middle, and distal were reported previously (Enes et al., 2023a). For the current exploratory analysis, we used only lateral thigh MT at 50% of femur length.

#### Training Sessions

Participants performed each supervised training session at the same time of the day to reduce the confounding effects of circadian rhythm fluctuations on perceptual responses. Participants performed lower limb RT sessions twice a week for 12 weeks. Prior to each training session, participants performed a general (5-min walking at 5 km∙h^−1^ on a treadmill and a light full-body dynamic stretching routine) and specific (2 sets of 6 and 3 repetitions with a self-selected load which the participants could perform for 10 and 6 repetitions maximum, respectively, throughout estimated repetitions in reserve) warm-up. Moreover, participants were instructed to refrain from any additional lower-limb RT or high-intensity exercise during the intervention.

All experimental groups performed the same RT scheme differing only in the weekly set number. The weekly-set progression was proportionally distributed among quadriceps exercises (barbell back squat, 45^o^ leg press and seated knee extension). For instance, to increase by three sets for one specific RT session, we followed the prescribed exercise order, increasing one set for the barbell back squat, one set for the 45^o^ leg press and one set for seated knee extension. In the final weeks of the study, the number of weekly sets for the barbell back squat, the 45^o^ leg press and seated knee extension totaled 8, 8 and 6 for the FS group, 14, 14, and 14 for the 4S group and 18, 18, and 16 for the 6S group, respectively. The loading scheme alternated between a lower repetition zone (6 to 8) and a higher repetition zone (10 to 12) for each exercise in the first and second training sessions, respectively. The intensity of effort was set at ~2 repetitions in reserve (RIR)-based ratings of perceived exertion, except the last set of each exercise that was taken to volitional concentric failure, defined as an inability to perform another concentric action while maintaining proper exercise form or until volitional termination of the set. When a participant did not reach the lower limit of the prescribed repetition zone or performed the previous set relatively easily, the load was reduced or increased accordingly for the next set. When a participant reached the volitional concentric failure in the last set of an exercise and exceeded the upper limit of the repetitions range, the load was increased for the next training session. These adjustments were made to ensure that participants stayed within the intended repetition range.

To reduce the possibility of subject dropouts and to maintain the consistency of their typical RT programs, we included lower-body posterior chain exercises in the RT program. The posterior chain exercises comprised two sets per exercise (the Romanian deadlift and seated knee flexion) with identical loading schemes. The exercise order was the barbell back squat, the 45^o^ leg press, seated knee extension, the Romanian deadlift, and seated knee flexion. Participants were allowed to train their upper body muscles as desired. Participants were afforded at least 2-min rest intervals between sets and exercises. Slight assistance by the research staff was provided when the participant was unable to achieve the targeted repetition zone, but only on the final 1–2 repetitions to ensure that the desired repetition range would be reached. The repetition tempo was 1 s for concentric actions and 2 s for eccentric actions with no pause during the transition phases between actions.

#### Perceptual Responses

Perceptual responses were assessed 15 min after the end of each RT session (Enes et al., 2023b; Scott et al., 2016); the sRPE was evaluated using the Borg CR-10 Scale and session Feelings of Pleasure/Displeasure (sFPD) were evaluated using the Affective Valence Scale (Foster et al., 2001; Hardy and Rejeski, 1989; Rejeski et al., 1987).

During the initial visits at baseline, participants were introduced to and familiarized with these scales, being instructed to report their overall effort throughout the whole training session, not only muscle-specific effort but also general effort (Christen et al., 2016; Scott et al., 2016; Singh et al., 2007), and their feelings of pleasure/displeasure related to training sessions, as well any questions were addressed during baseline testing.

We gauged the sRPE by asking “How was your training session?” (Christen et al., 2016; Foster et al., 2001). The sRPE assessed by the Borg’s scale provides a score from 0 to 10 (or more, if the participant never experienced similar effort previously) related to the global effort of the RT session. For further analysis, we calculated the sRPE multiplied by the volume load (sRPE-VL) or by the number of sets performed in the training session (sRPE-S) (Foster, 1998; Foster et al., 2001; Haddad et al., 2017; Helms et al., 2020). Moreover, the density of the training session was calculated by dividing sRPE-VL and sRPE-S by training duration (D-VL and D-S, respectively) (Helms et al., 2020). Monotony and strain values were assessed from sRPE-S. Monotony was calculated by weekly average of sRPE-S divided by its standard deviation and strain was calculated by the sum of sRPE-S multiplied by their monotony values related to the specific week of analysis (Foster, 1998; Helms et al., 2020).

The Affective Valence Scale provides a score related to FPD in an 11-point bipolar scale, ranging from −5 to +5 (0 being neutral), with negative to positive numbers representing unpleasurable and pleasurable feelings, respectively (Ekkekakis et al., 2011; Hardy and Rejeski, 1989). The sFPD were assessed by individually asking participants “How was your workout?” and they indicated their perceptual responses on the scale. The weekly averages of the perceptual responses were used for further analysis. All of these variables were expressed in arbitrary units (a.u.). The VL progression slopes (expressed in %) were calculated as: the week of interest (*x*) = [((VL *x* – VL week 1)/VL week 1)*100].

### Statistical Analysis

Data normality of model residuals and homogeneity of variances were determined by Shapiro-Wilk and Levene’s tests, respectively. Data were expressed as mean ± standard deviation (SD). A linear mixed-model analysis was used to detect any potential group-by-time interaction for each dependent variable, assuming time and group as fixed factors and subjects as a random factor. A Bonferroni test correction was applied when the F value was significant for multiple comparisons. The Pearson's product-moment correlation coefficient was used to analyze the association among volume load progression slopes, MT, and perceptual variables, as two-tailed tests. The Pearson’s correlation was interpreted as negligible (r = 0.00–0.10), weak (r = 0.10–0.39), moderate (r = 0.40–0.69), strong (r = 0.70–0.89) or very strong (r = 0.90–1.00). The significance level was established *a priori* at *p* < 0.05. All analyses were conducted in SPSS software version 25.0 (IBM Corp. Released 2017. IBM SPSS Statistics for Windows, Version 25.0. Armonk, NY: IBM Corp.).

## Results

No group-by-time interactions were observed for MT values (F_(2,28)_ = 2.172; *p* = 0.133), only a main effect of time (F_(1,28)_ = 24.145); *p* < 0.0001).

**Figure 2:**
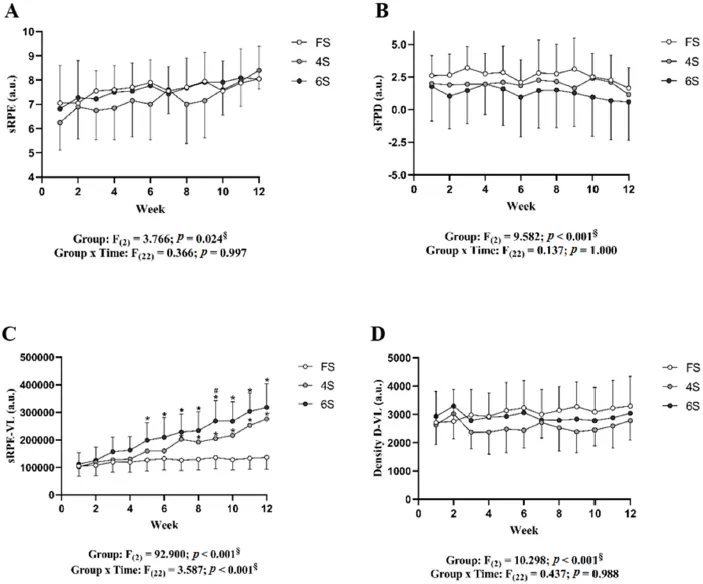
Perceptual responses in different progressive RT volume models. Note: Panel A: the session rating of perceived exertion (sRPE) over 12 weeks of training. Panel B: the session affective valence scale (FPD) over 12 weeks of training. Panel C: the session rating of perceived exertion (sRPE) calculated by total training volume over 12 weeks of training. Panel D: density calculated by the total training volume over 12 weeks of training. Note: * significant difference from the FS group; ^#^ significant difference from the 4S group; ^§^ main effect (group) or significant interaction (time x group); FS: fixed sets; 4S: four sets progression training volume; 6S: six sets progression training volume

Figure 2 depicts the perceptual variables over the 12-week period among the experimental groups. There was a time effect (F_(11,336)_ = 2.77, *p* = 0.002) in weeks 11 and 12 where greater sRPE responses were observed than in the other weeks (*p* < 0.05), and also a group effect (Figure 2A) for the sRPE, as the 4S group showed lower sRPE responses than the FS group (*p* = 0.046) and a trend for lower sRPE responses than in the 6S group (*p* = 0.061), but no differences between FS and 6S groups (*p* = 0.999). Figure 2B shows that sFPD remained constant over the intervention, with more pleasant values reported for the FS group compared to the 6S group (*p* < 0.001). Moreover, as depicted in Figure 2A and B, no significant group-by-time interaction was detected for the sRPE and sFDP.

Figure 2C depicts the sRPE-VL during the experimental protocol. There was a significant interaction (time and group; *p* < 0.001), indicating that 4S (5^th^ to 12^th^ weeks) and 6S (7^th^ to 12^th^ weeks) groups showed higher sRPE-VL values compared to the FS group. In addition, only in the 9^th^ week did the 6S group present higher sRPE-VL values than the 4S group (*p* = 0.017). Finally, the significant main group effect indicated that 4S and 6S groups presented higher sRPE-VL values when compared to the FS group (*p* < 0.001). Similarly, the 6S group presented higher sRPE-VL values when compared to the 4S group (*p* < 0.001). Figure 2D indicates that, in general, D-VL was higher in 6S (*p* = 0.004) and FS (*p* < 0.001) groups when compared to the 4S group. There was no significant interaction for the D-VL variable.

**Figure 3:**
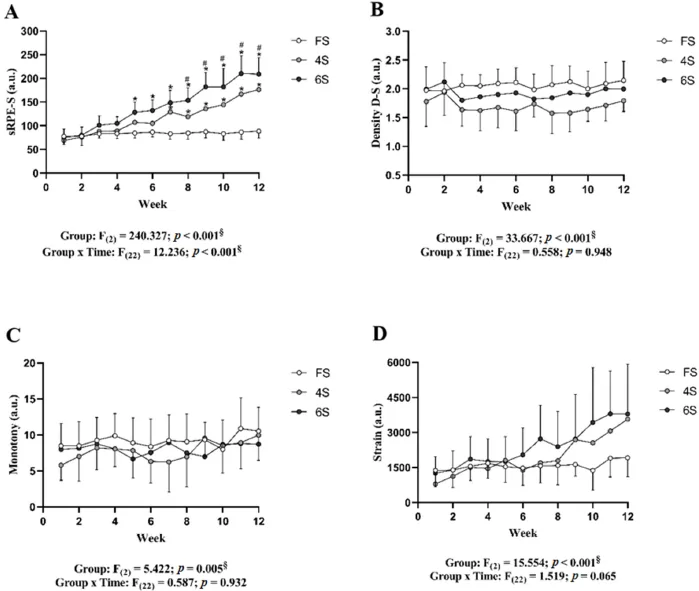
Perceptual responses analyzed based on the weekly-set number. Panel A: session rating of perceived exertion (RPE) calculated by sets performed over 12 weeks of training. Panel B: density calculated by sets performed over 12 weeks of training. Panel C: training monotony over 12 weeks. Panel D: training strain over 12 weeks. Note: * significant difference from the FS group; ^#^ significant difference from the 4S group; ^§^ main effect (group) or significant interaction (time x group); FS: fixed sets; 4S: four sets progression training volume; 6S: six sets progression training volume.

Figure 3 shows the perceptual variables derived from the weekly-set number performed throughout the 12-week period. Figure 3A depicts a significant interaction (*p* < 0.001) and a main effect of group (*p* < 0.001). Post-hoc comparisons revealed that in the 5^th^ and 6^th^ weeks, sRPE-S values were higher in the 6S group compared to the FS group (*p* < 0.001). In the 7^th^ week, sRPE-S values were higher in both the 4S and 6S groups compared to the FS group (*p* < 0.001). From the 8^th^ to the 12^th^ week, the groups differed significantly (*p* < 0.001), with the 6S group showing greater values than the 4S group and the 4S group showing greater values than the FS group (6S > 4S > FS). Figure 3B indicates the main effect of the group, with the FS protocol displaying a higher D-S value than the 4S group. Figure 3C revealed a significant effect of monotony, where FS and 6S groups presented higher values compared to the 4S group. Finally, Figure 3D shows an effect of strain, where a higher progression in the number of weekly sets corresponded to more significant strain (6S > 4S > FS).

Table 1 displays the correlation values between the VL progression slope with changes in MT [absolute (mm) and relative (%)] and the perceptual training variables of 12 weeks (sRPE, sFPD, Monotony-S, and Strain-S). The results indicated a positive association between Slope VL 3 with MT absolute (r = 0.362) and MT relative (r = 0.392). The Slope VL 5 also was positively associated with MT relative (r = 0.358). Considering the perceptual variables during the 12 weeks of experimental protocols and their respective correlation with the Slope VL, we found that FPD in week 3 were negatively associated with Slope VL 2 (r = −0.379). In addition, Monotony-S in week 9 was negatively associated with Slope VL 8 (r = −0.373), and Strain-S in week 11 was positively associated with Slope VL 10 (r = 0.448).

**Table 1:** Correlation between Slope VL, changes in muscle thickness (%), and 12 weeks of perceptual variables.

	Change (%)		Week				
	MT Raw	MT Relative	sRPE	sFPD	Monotony	Strain	
Slope VL 1	0.161	0.160	0.164	−0.254	−0.048	0.026	Week 2
Slope VL 2	0.240	0.279	−−0.117	−0.379*	−0.195	0.013	Week 3
Slope VL 3	0.362*	0.392*	0.035	−0.281	−0.297	−0.039	Week 4
Slope VL 4	0.342	0.344	−0.006	−−0.333	−0.187	0.129	Week 5
Slope VL 5	0.345	0.358*	−0.002	−0.167	−0.106	0.198	Week 6
Slope VL 6	0.269	0.273	−0.030	−0.198	−0.167	0.230	Week 7
Slope VL 7	0.222	0.234	−0.158	−0.160	−0.264	0.078	Week 8
Slope VL 8	0.277	0.292	−0.115	−0.227	−0.373*	0.172	Week 9
Slope VL 9	0.289	0.310	0.028	−0.233	−0.152	0.254	Week 10
Slope VL 10	0.291	0.307	−0.036	−0.168	−0.206	0.448*	Week 11
Slope VL 11	0.298	0.313	−0.034	−0.162	−0.278	0.350	Week 12

Note: MT: muscle thickness; VL: volume load; sRPE: rating of perceived exertion in a training session; sFPD: the feeling of pleasure/displeasure in a training session. * shows a significant correlation (p < 0.05)

Table 2 depicts correlations between the changes in MT (absolute and relative) and the sRPE variable for 12 weeks. The results indicated a positive association between the sRPE in week 2 with MT absolute (r = 0.396) and MT relative (r = 0.400).

**Table 2:** Correlation between muscle thickness (raw and relative) and the session rating of perceived exertion (sRPE) in 12 weeks.

	MT Raw (%)	MT Relative (%)
sRPE Week 1	0.210	0.233
sRPE Week 2	0.396^*^	0.400^*^
sRPE Week 3	0.284	0.275
sRPE Week 4	0.195	0.188
sRPE Week 5	0.182	0.182
sRPE Week 6	0.185	0.186
sRPE Week 7	0.051	0.059
sRPE Week 8	0.248	0.221
sRPE Week 9	0.223	0.210
sRPE Week 10	0.331	0.341
sRPE Week 11	0.296	0.302
sRPE Week 12	0.255	0.242

Note: MT: muscle thickness; sRPE: rating of perceived exertion in a training session; * indicates a significant correlation (p < 0.05)

## Discussion

We investigated the effects of increasing the weekly set number targeting the quadriceps femoris on perceptual responses in resistance-trained males and hypothesized a potential linear relationship between RT volume and perceptual responses due to increases in the external training load. We also investigated whether VL metrics and MT correlated with perceptual responses during a RT program. While different weekly-set progressions influenced the perceptual responses, sRPE-volume derived metrics, sFPD, monotony, and strain did not demonstrate any consistent and meaningful relationship with changes in the volume load, contrary to our hypothesis. Indeed, among the perceptual variables, the only outcome associated with volume was strain, wherein higher weekly set progressions corresponded with higher strain in accordance to our hypothesis, although the meaningfulness was weak. In addition, there were no meaningful relationships between muscle thickness and either external load variables (volume load progression slopes) or perceptual responses (sRPE).

The 4S condition elicited a slightly lower absolute sRPE than FS and 6S conditions, although the meaningfulness of the mean differences remains uncertain. Contrary to this, Aube et al. (2022) and Enes et al. (2024) found comparable sRPE values among different RT volumes in longitudinal interventions, as determined by the weekly set number and carried out at similar proportional intensities of effort. Volume load and sRPE trends of the current study are less easily reconciled with previous findings. While increases in VL across FS, 4S, and 6S conditions especially in the later weeks of the intervention and concomitant increases in the sRPE align with those of Lodo et al. (2012) and Genner and Weston (2014), where found positive correlations between the sRPE and VL were found, these studies were both acute designs. On the other hand, Aube et al. (2022) found no changes in the sRPE, with participants performing a fixed number of RT sets at the same intensity of effort for the duration of the study. Noteworthy, however, unlike the present study where the lowest prescribed volume was 22 weekly sets, the highest weekly-set volume studied by Aube et al. (2022) was 24 sets. Thus, it is possible that a non-linear relationship exists between volume and the sRPE above a certain threshold. Nevertheless, and given the totality of evidence, intensity of effort, i.e., proximity to failure, may be a greater determinate of the sRPE than VL, particularly given the equivocal relationship between VL and the sRPE (Genner and Weston, 2014; Hiscock et al., 2018; Lodo et al., 2012; Martorelli et al., 2021).

While the current study found that sFPDs did not change significantly within groups despite increasing set numbers (4S and 6S) and volume loads, the consistent sFPD ratings are similar to previous studies that found no affective valence differences between different RT volumes as meaured by the number of sets (Aube et al., 2022; Enes et al., 2024) or VL, regardless of the periodization model (Conlon et al., 2015). Indeed, affective feelings are associated with the participants’ motivation and adherence to a training program (Andersen et al., 2022; Ekkekakis et al., 2011). Our findings suggest that progressively increasing training volume might not compromise participants’ engagement or adherence to the RT program within a 12-week period. However, given the design of the current study and the slightly lower sFPD for the 6S compared to the FS condition, further research is needed on the effects of progressive volume increases on affective feelings towards RT over a longer timeframe, since the 4S condition was similar to FS and 6S conditions.

Given the consistent sRPE values in the current study, findings that higher strain scores correspond with higher weekly set progressions would be expected. Initially, it appeared that these results differed from those of Conlon et al. ( 2015) who found strain to be unaffected by VL across different RT periodization approaches. However, weekly changes in VL compared to baseline did not appear to influence strain in the current study as well as the current study also found monotony to be unrelated to VL or changes in VL, in accordance to Conlon et al. (2018). Importantly, both previous studies (Conlon et al., 2015, 2018) investigated a RT program in older adults, limiting comparisons between our findings. In addition, although strain scores were usually associated to lack of training-induced adaptations (Clemente et al., 2019; Conlon et al., 2015; McGuigan and Foster, 2004; Scott et al., 2016), this was not detected based on our findings, similarly to Conlon et al. (2015).

Despite the weekly set progression differences among conditions (Aube et al., 2022; Enes et al., 2023a), it remains unclear whether the isolated positive associations between Slope VL 3 and VL 5 between MT and sRPE in week 2 (absolute and relative) can be attributed to deleterious effects with participants’ adaptation to the varied phases (washout, familiarization, etc.). Additionally, further research is needed to better elucidate the relationship between monitoring previous training volume (Aube et al., 2022; Enes et al., 2023a) and the influence of differing VL and set progression schemes on muscular hypertrophy, as well as to better understand the interactions among MT, VL and perceptual responses (sRPE, sFPD, monotony, and strain).

Our study has some limitations that need to be addressed when attempting to draw practical inferences from the findings. Initially, given what is known about the influence of dietary protocols on muscle accretion, individual nutritional tracking discrepancies could have negatively influenced hypertrophic outcomes. Moreover, to mitigate erroneous effects of previous training volume on musculoskeletal and perceptual responses, we implemented a two-week washout phase followed by a familiarization phase. However, we are unsure whether and to what degree the washout weeks facilitated the subject’s ability to fully acclimate to the novel training volume stimulus. Additionally, we are unsure whether the subject-specific variations in previous training volume were influenced by the group standardized 22 sets per week and how this compares against differing volume load progression schemes. Expanding on the current study design, despite the addition of posterior lower-limb exercises, hypertrophic outcomes were only measured in the lateral aspects of the thigh, therefore the present MT findings cannot be extrapolated to other various anterior and posterior aspects of the lower-limb or upper-limb musculature. To assist adherence to the training program, participants were allowed to train their upper body as desired. However, upper body training was not controlled; therefore we cannot determine whether it may have influenced our findings. Lastly, the findings can only be generalized to resistance-trained males, thereby limiting inferences to other populations.

## Conclusions

In summary, our findings indicate that different weekly-set progressions lead to differential responses in perceptual responses, but without associations between MT and volume-derived metrics in resistance-trained males. Coaches and practitioners monitoring perceptual responses such as the sRPE and its derived metrics, as well as sFPD, can assess the impact of weekly set volume progressions on individual perceptual responses to the RT program, given the low-cost and non-invasive nature of perceptual responses. As sRPE metrics and sFPD differed substantially, especially in higher-volume groups, adjustments such as modifying volume metrics, may be required to prevent excessive fatigue and/or sustain adherence, especially for longer time frames than in the present study. Moreover, since strain followed a dose-response, tailoring weekly set volume based on individual tolerance and recovery capacity might optimize training adaptations, however, further studies are warranted. However, the perceptual responses may not be a meaningful predictor of changes on MT at the mid portion of the lateral thigh within a 12-week period, therefore coaches and practitioners can use the perceptual responses to focus on other aspects such as fine-tuning adjustments and support long-term adherence to the RT program.
